# Supplementary material for: The Neglected Angio-Neurotrophic Parasite Gurltia paralysans (Nematoda: Angiostrongylidae): Northernmost South American Distribution, Current Knowledge, and Future Perspectives
Source: Pathogens. 2021 Dec 9;10(12):1601. doi: 10.3390/pathogens10121601 (PMC8707030; doi:10.3390/pathogens10121601)
Supplement: Supplementary file 1 [file pathogens-10-01601-s001.zip › pathogens-1493473-supplementary.pdf]

Table S1. Gastropod species as potential intermediate hosts for *G. paralysans* in Colombia.

Taxon	Habitats [1]	Distribution [2]
**Neritidae**		
Neritina laetissima	B, F	P
Neritina punctulata	B, F	C
Neritina virginea	B, F	C
**Ampullariidae**		
Asolene crassa	F	C, M-C
Marisa cornuarietis	F	A, C, M-C, O
Pomacea bridgesii	F	A
Pomacea canaliculata	F	A
Pomacea dolioides	F	O
Pomacea flagellata	F	C, M-C
Pomacea glauca	F	C, M-C
Pomacea haustrum	F	M-C
Pomacea maculata	F	A, O
Pomacea oblonga	F	M-C, O
Pomacea palmeri	F	M-C
Pomacea papyracea	F	A, O
Pomacea superba	F	C, M-C
Pomacea urceus	F	A, O
**Thiaridae**		
Aylacostoma sp.	F	O
Melanoides tuberculata	F	C, M-C, O
Tarebia granifera	F	C, M-C
**Cochliopidae**		
Andesipyrgus sketi	F/Tg	M-C
Aroapyrgus colombiensis	F	M-C
Lithococcus venustus	F	P
Pyrgophorus parvulus	F	C, M-C
**Lymnaeidae**	F	
Pseudosuccinea columella	F	C, M-C, O
Galba cousini	F	M-C, O, P
Galba truncatula	F	M-C
**Physidae**		
Physa acuta	F	C, M-C
Stenophysa marmorata	F	C, M-C, O
**Planorbidae**		
Biomphalaria amazonica	F	A
Biomphalaria edisoni	F	M-C
Biomphalaria kuhniana	F	M-C, O
Biomphalaria pallida	F	C
Biomphalaria peregrina	F	M-C, O
Biomphalaria straminea	F	M-C, O
Drepanotrema anatinum	F	M-C, O
Drepanotrema cimex	F	M-C

Drepanotrema depresissimum	F	M-C
Ferrisia irrorata	F	M-C
Gundlachia radiata	F	C, M-C
Planorbella duryi	F	M-C
Uncancylus concentricus	F	C, M-C
**Cyclophoriidae**		
Filocyclus delphinulus	T	¿CO?
Calacyclotus atratensis	T	¿CO?
**Megalomastomidae**		
Aperostoma sp.	T	M-C, C
**Neocyclotidae**		
Daronia sp.	T	M-C
Calaperostoma	T	P
Poteria sp.	T	M-C, C
Neocyclotus sp.	T	¿CO?
**Achatinidae**		
Achatina sp.	T	¿CO?
Rhodea sp.	T	M-C
Subulina sp.	T	M-C, C, O, P
Synapterpes sp.	T	M-C, P
**Agriolimacidae**		
Deroceras sp.	T	M-C, P
**Arionidae**		
Arion sp.		M-C
**Boettgerillidae**		
Boettgerilla compressa	T	A, M-C
Boettgerilla pallens	T	M-C
**Bulimulidae**		
Auris sp.	T	O
Bulimulus sp.	T	M-C, C
Drymaeus sp.	T	A, M-C, C
Naesiotus sp.	T	M-C, P
**Simpulopsidae**		
Simpulopsis sp.	T	
**Amphibulimidae**		
Dryptus sp.	T	M-C, C
Plekocheilus sp.	T	O, M-C, C
Stenostylus sp.	T	¿CO?
**Megaspiridae**		
Thaumastus sp.	T	P¿CO?
**Orthalicidae**		
Corona sp.	T	A
Hemibulimus dennisoni	T	M-C
Hemibulimus excisus	T	M-C
Orthalicus sp.	t	M-C, C
Porphyrobaphe sp.	T	P

Sultana sp.	T	M-C
**Labyrinthidae**		
Isomeria sp.	T	M-C
Labyrinthus sp.	T	A, M-C, C, P
**Pleurodontidae**		
Pleurodonte sp.	T	P
**Solaropsidae**		
Solaropsis sp.	T	M-C, O
**Cerioniidae**		
Cerion sp.	T	C
**Charopidae**		
Radiodiscus sp.	T	M-C
**Clausiliidae**		
Clausilia sp.	T	M-C
Columbinia sp.	T	P
Nenia sp.	T	M-C, C
**Euconuliidae**		
Habroconus sp.	T	P
**Ferrussaciidae**		
Cecilioides sp.	T	C, O
**Helicidae**		
Helix sp.	T	M-C
**Epiphragmophoridae**		
Epiphragmophora sp.		
**Limacidae**		
Lehnmania sp.	T	M-C
Limax sp.	T	M-C
**Strophocheilidae**		
Megalobulimus sp.	T	C
**Urocoptidae**		
Microceramus sp.	T	C, O
**Milacidae**		
Milax sp.	T	M-C
**Phylomicidae**		
Philomycus sp.	T	¿CO?
**Polygyridae**		
Giffordius sp.	T	M-C, C, O
**Punctidae**		
Paralaoma sp.	T	M-C
**Pupillidae**		
Pupoides sp.	T	C
**Sagdidae**		
Lacteoluna sp.	T	C, O
**Scolodontidae**		
Hirtudiscus sp.	T	M-C
**Spiraxidae**		
Euglandina sp.	T	M-C, C, P

Pseudosubulina sp.	T	¿CO?
**Streptaxidae**		
Streptaxis sp.	T	M-C, C, O
Streptostele sp.	T	M-C
**Strobilopsidae**		
Strobilops sp.	T	C
**Strophocheilidae**		
Strophocheilus sp.	T	M-C, C, O
**Subulinidae**		
Leptinaria sp.	T	C
**Pyramidellidae**		
Pyramidella sp.	T	M-C
**Opeatinae**		
Opeas sp.	T	C, O
**Succineidae**		
Omalonyx sp.	T	A
Succinea sp.	T	M-C, C, O
**Scolodontidae**		
Guestieria sp.	T	M-C
Happia sp.	T	M-C
Miradiscops sp.	T	C, O
Systrophia sp.	T	M-C, A, C, O, P
**Thysanophoridae**		
Thysanophora sp.	T	C, O
**Valloniidae**		
Vallonia sp.	T	C
**Pristilomatidae**		
Hawaiia sp.	T	M-C
Vitrea sp.	T	M-C
**Gastrocoptidae**		
Gastrocopta sp.	T	C
**Valloniidae**		
Pupisoma sp.	T	C
**Vertiginidae**		
Vertigo sp.	T	C, O
**Xanthonychidae**		
Leptarionta sp.	T	¿CO?
**Oxychilidae**		
Oxychilus sp.	T	M-C

[1] Habitats: Fresh water (F), Brackish water (B), Terrestrial (T), Tg (Troglobio). [2] Watershed distribution: A (Amazonian), C (Caribbean), M-C (Magdalena-Cauca), O (Orinoquian), P (Pacific).
